# Comparative Study of Lectin Domains in Model Species: New Insights into Evolutionary Dynamics

**DOI:** 10.3390/ijms18061136

**Published:** 2017-05-25

**Authors:** Sofie Van Holle, Kristof De Schutter, Lore Eggermont, Mariya Tsaneva, Liuyi Dang, Els J. M. Van Damme

**Affiliations:** 1 Department of Molecular Biotechnology, Faculty of Bioscience Engineering, Ghent University, Coupure Links 653, 9000 Ghent, Belgium; Sofie.VanHolle@UGent.be (S.V.H.); Kristof.DeSchutter@UGent.be (K.D.S.); Lore.Eggermont@UGent.be (L.E.); Mariya.Tsaneva@UGent.be (M.T.); Liuyi.Dang@UGent.be (L.D.); 2 Department of Crop Protection, Faculty of Bioscience Engineering, Ghent University, Coupure Links 653, 9000 Ghent, Belgium

**Keywords:** lectin, carbohydrate, evolution, phylogeny, protein domain

## Abstract

Lectins are present throughout the plant kingdom and are reported to be involved in diverse biological processes. In this study, we provide a comparative analysis of the lectin families from model species in a phylogenetic framework. The analysis focuses on the different plant lectin domains identified in five representative core angiosperm genomes (*Arabidopsis* *thaliana*, *Glycine max*, *Cucumis sativus*, *Oryza sativa* ssp. *japonica* and *Oryza sativa* ssp. *indica*). The genomes were screened for genes encoding lectin domains using a combination of Basic Local Alignment Search Tool (BLAST), hidden Markov models, and InterProScan analysis. Additionally, phylogenetic relationships were investigated by constructing maximum likelihood phylogenetic trees. The results demonstrate that the majority of the lectin families are present in each of the species under study. Domain organization analysis showed that most identified proteins are multi-domain proteins, owing to the modular rearrangement of protein domains during evolution. Most of these multi-domain proteins are widespread, while others display a lineage-specific distribution. Furthermore, the phylogenetic analyses reveal that some lectin families evolved to be similar to the phylogeny of the plant species, while others share a closer evolutionary history based on the corresponding protein domain architecture. Our results yield insights into the evolutionary relationships and functional divergence of plant lectins.

## 1. Introduction

*Arabidopsis thaliana* was the first plant to have its genome completely sequenced in 2000 [1]. More than 60 plant genomes have been published to date, representing species from different lineages of Viridiplantae. The wealth of available completely sequenced plant genomes presents an unrivaled opportunity for comparative analysis and will continue to reveal new aspects of genome biology and evolution. For example, analysis of gene family expansion and evolution across species is employed to identify genes with a shared evolutionary origin. In general, these homologous genes also demonstrate comparable biological functions [2]. The classification of protein-coding genes to known gene families is based on protein sequence similarity. However, a considerable number of genes are “orphans” because they lack homology to any known protein [3]. To date, the extent and role of gene families in plants has only been partially addressed and many gene families remain to be investigated [2].

Protein domains are structurally conserved and represent functionally independent components of proteins. The domains themselves have evolved from shorter structural units (e.g., repeats) or by the association of small folding motifs [4,5]. These dominant structural units of proteins are evolutionarily well preserved across taxa [6]. Furthermore, evolutionary events such as duplication, fusion, fission, domain gain and domain loss drive protein domain rearrangements of single-domain proteins and act on the evolution and expansion of multi-domain proteins [7,8,9,10]. Not surprisingly, the number of multi-domain architecture families is growing exponentially by the rearrangement and/or combination of existing domains. Conversely, new single-domain architecture families are arising slowly [9,11]. Single-domain proteins are therefore more likely to be shared by large groups of species while multi-domain proteins display unique architectures that are more specific, thus accounting for species diversity [11,12]. The formation of multi-domain proteins is an important evolutionary process that gives rise to proteins with new functions explained by their adaptive benefits in response to environmental challenges [13,14,15].

The plant lectin family encompasses all proteins that selectively recognize and bind to specific carbohydrate structures that occur in a free form or as part of glycoconjugates, such as glycoproteins and glycolipids. The binding of lectins to glycan structures is reversible and does not alter the structure of the glycan moiety [16]. This protein–carbohydrate interaction is involved in a myriad of important processes in the plant such as disease resistance, symbiosis or self-incompatibility. According to their conserved carbohydrate-recognition domain, plant lectins can further be divided into 12 distinct subfamilies: the *Agaricus bisporus* agglutinin family, the amaranthins, the homologs of class V chitinases (CRA), the cyanovirin family, the *Euonymus europaeus* lectin (EUL) family, the *Galanthus nivalis* agglutinin (GNA) family, the hevein family, the jacalin-related lectin (JRL) family, the legume lectin family, the lysin motif (LysM) family, the *Nicotiana tabacum* agglutinin (Nictaba) family and the ricin B lectin family [17]. Proteins belonging to a particular plant lectin family are evolutionarily related and the corresponding carbohydrate-recognition domain is defined by its amino acid sequence and the structure of the binding site. Nevertheless, proteins from the same plant lectin family can recognize different carbohydrate structures. These promiscuous carbohydrate-binding sites make it difficult to predict the biological properties of homologous proteins [18,19,20]. Aside from the classification of plant lectins based on their conserved carbohydrate-recognition domain, lectins can also be grouped according to their expression pattern and subcellular localization. Originally, plant lectins were identified in seeds and the vegetative storage tissues of plants. These lectin genes are constitutively expressed and the corresponding proteins are often synthesized with a signal peptide, guiding them to the secretory pathway. In addition to their role as a storage protein, some of these lectins also play a role in plant defense against pathogens and/or herbivory [21]. More recently, a new class of lectin genes was identified, comprising of inducible lectin genes. Under normal conditions, these proteins are only present at basal levels, but their expression can be induced in response to particular biotic or abiotic stimuli. In contrast to the classical lectins described above, these stress-inducible lectins are located in the nucleocytoplasmic compartments of the plant cell. Some nucleocytoplasmic lectins have been investigated in detail, and are proposed to be involved in stress signaling [22,23,24,25,26].

Although lectins from plants and other species have been an important topic of research for years, most studies dealt with the identification and expansion of a particular lectin family in one species, or specifically focused on one type of lectin motif and its distribution in the plant kingdom [27,28,29,30,31,32,33,34,35,36]. To date, there have only been a few publications that report on the distribution of multiple lectin motifs across plant species. Recently, genome wide studies of lectin motifs have been performed in *Glycine max* (soybean) [37], *Cucumis sativus* (cucumber) [38], *Arabidopsis thaliana* (Arabidopsis, thale cress) [39] and *Morus notabilis* (mulberry) [28]. In this study, a comparative analysis was made of the lectin motifs in several model plants, in particular three important dicot species (soybean, cucumber, Arabidopsis) and the monocots *Oryza sativa* ssp. *japonica* and *Oryza sativa* ssp. *indica*. The distribution of lectin domains was examined and the phylogenetic relationships of the different plant lectin motifs were investigated. Our comparative study revealed that additional protein domains have recurrently been integrated into lectins from multiple plant species. Using a phylogenetic approach, we demonstrated the differential modes of evolutionary innovation and observed compelling differences between the lectin families.

## 2. Results and Discussion

### 2.1. Most Plant Lectin Domains Are Widely Distributed in Arabidopsis, Soybean, Cucumber and Rice

To gain insight into the evolution and diversity of lectin protein architectures across plants, the genomes of *Arabidopsis thaliana*, *Glycine max*, *Cucumis sativus*, *Oryza sativa* ssp. *japonica* and *Oryza sativa* ssp. *indica* were scanned for the presence of genes encoding plant lectin domains. All predicted lectin domains and any additional protein domains were identified using InterProScan. Altogether, 1337 lectin domain containing sequences were identified, belonging to 10 of the 12 plant lectin families known today (Table 1). Lectin genes belonging to the *Agaricus bisporus* agglutinin family or the cyanovirin lectin family were not identified in the species under study. Remarkably, the presence of amaranthin lectin genes was confined to the cucumber genome, suggesting that the latter three groups of plant lectins are not widespread in angiosperms. Indeed, cyanovirin homologs were only identified in bacteria, filamentous ascomycetes and the fern *Ceratopteris richardii* [40]. Similarly, homologs of the *Agaricus bisporus* agglutinin have only been described in fungi and the liverwort *Marchantia polymorpha* [41,42]. Members of the amaranthin family have been identified in lower plants (*Selaginella moellendorffii*) as well as in monocots (maize, barley, wheat, etc.) and in several dicot plants from different families (sugar beet, grape, melon, flax, etc.); demonstrating that this lectin domain is widespread, yet not omnipresent [43,44].

The total number of identified lectin genes is highly divergent (146-368) when comparing different species, but no correlation was found between the genome size and the number of lectin genes (Table S1). Considering the number of protein-coding transcripts identified for each species, the ratio of lectin genes varies between 0.42 and 0.62. The Arabidopsis and rice genome encompass the highest percentage of lectin genes. It has been demonstrated that whole genome duplications (WGDs) contributed to species diversification and gene expansion [45,46,47,48]. All angiosperms have undergone at least two ancient rounds of WGDs which took place in the ancestor of angiosperms and of seeds plants [49]. Subsequently, the Arabidopsis lineage underwent three additional polyploidy events [50], similar to the rice and soybean genome, which independently also have undergone three rounds of WGDs [51,52,53,54,55]. The cucumber genome on the other hand, has only experienced one polyploidy event that is shared by all core eudicots and thus represents a more ancestral genome [56,57]. In spite of the differences in the number of WGDs in the four plant species under study, there is no correlation between the occurrence of multiple WGDs and the total number of lectin genes in the different species. Analysis of the distribution of the lectin domains (LysM, GNA, JRL, legume lectin, Nictaba, hevein and ricin B domain) in the PLAZA database [58] revealed the presence of these domains in a wide range of species from lower plants (*Physcomitrella patens* and *Amborella trichopodia*) to monocots and dicots.

A comparative analysis of the size of the different plant lectin families (Table 1), demonstrates that the GNA and legume lectin homologs are most abundant. Apart from Arabidopsis, the GNA family is the largest lectin family in all species representing 30–45% of all lectin sequences. In rice and soybean, the GNA and legume lectin family together account for more than 70% of all lectin genes. The third largest family is the LysM family (12.8%) in soybean, the Nictaba family (13.7%) in cucumber and the jacalin family (9.2%) in rice. In Arabidopsis, the jacalin family (23.1%) is the second largest family after the legume lectin family (25.0%), and the GNA family (22.7%) completes the top three. In this context, PLAZA analyses indicate that a combination of tandem and segmental duplications are responsible for the expansion of these lectin families in Arabidopsis, soybean and rice.

### 2.2. Domain Organization of Lectin Genes in Arabidopsis, Soybean, Cucumber and Rice

Protein domains are the building blocks of all proteins, and the combination of particular protein domains defines protein functionality [59]. Using a combination of InterProScan5 and hidden Markov models, all annotated protein domains were identified in the putative lectin sequences. Table 2 illustrates the most common domain architectures present in Arabidopsis, soybean, cucumber and rice. An enumeration of all identified domain architectures in these species is presented in Table S2.

Protein sequences consisting of multiple lectin domains were found in most lectin families (EUL, hevein, jacalin, legume lectin, LysM, Nictaba and ricin B); though no combinations of different lectin domains within one putative lectin sequence were retrieved. Furthermore, the lectin domains arrayed in tandem can also be linked to additional, unrelated protein domains. Examples are the amaranthin/amaranthin/aerolysin combination in cucumber and the LysM/LysM/protein kinase domain combination that is present in all the species under study. The EUL lectin domain is the only lectin domain that is not present in combination with other annotated protein domains, though proteins composed of two EUL domains arrayed in tandem were identified in rice. These results support previous studies which concluded that in addition to *Physcomitrella patens* and *Selaginella moellendorffii*, mainly monocots comprise EUL domains arrayed in tandem while the single-domain proteins are present in both monocot and dicot species [60,61]. 

Our analysis revealed that almost all lectin domains can occur as single-domain proteins. However, it should be mentioned that in those proteins, the full-length protein sequence does not always consist of the lectin domain alone. The additional N- or C-terminal sequences are unrelated to the lectin domain, but do not correspond to any known protein domain. The majority of the identified domain architectures represent multi-domain proteins, consisting of one or more lectin domain(s), in combination with other annotated protein domains. Glycosyl hydrolase (GH) domains were found in combination with the hevein domain (GH19), the legume lectin domain (GH1 or GH17) and the ricin B lectin domain (GH5 or GH27). Similarly, the F-box domain was identified in combination with the jacalin, LysM and Nictaba domains in multiple species. Another reoccurring protein domain found in all species is the protein kinase (PK) domain. This protein domain is associated with the CRA, GNA, jacalin, Nictaba, LysM and legume lectin domains in various species. 

Furthermore, a lot of unique domain combinations were identified, comprising a lectin domain and one or more additional protein domain(s). The presence of these protein domain architectures is predominantly limited to a particular species, supporting previous observations that long multi-domain architectures tend to be more species-specific. However, a large majority of all plant domain arrangements was shown to be present in all linages; as illustrated in Table 2. Previous investigations revealed that if a protein domain is present in a particular species, there tend to be multiple genes encoding proteins in this domain [12,62]. Based on Table 1, this is also applicable to lectin encoding genes with the exception of the EUL family, of which there is only one gene present in the genomes of Arabidopsis and cucumber. Moreover, these studies showed that species without a recent WGD have lower rates of protein rearrangement. This is in agreement with our data as the cucumber genome (the only species in our study without recent WGDs) encompasses the lowest number of species-specific domain combinations (Table S2). 

### 2.3. Phylogenetic Relationships and Biological Significance

A selection of lectin domains and some reccurring combinations of lectin domains and particular non-lectin protein domains were investigated in more detail. Evolutionary relationships between the protein domains from the different species were investigated by building maximum likelihood trees with RAxML. The results of the phylogenetic analyses and the biological importance of the protein domain combinations are discussed below.

#### 2.3.1. Ricin B and GH Domains

**Figure 1:**
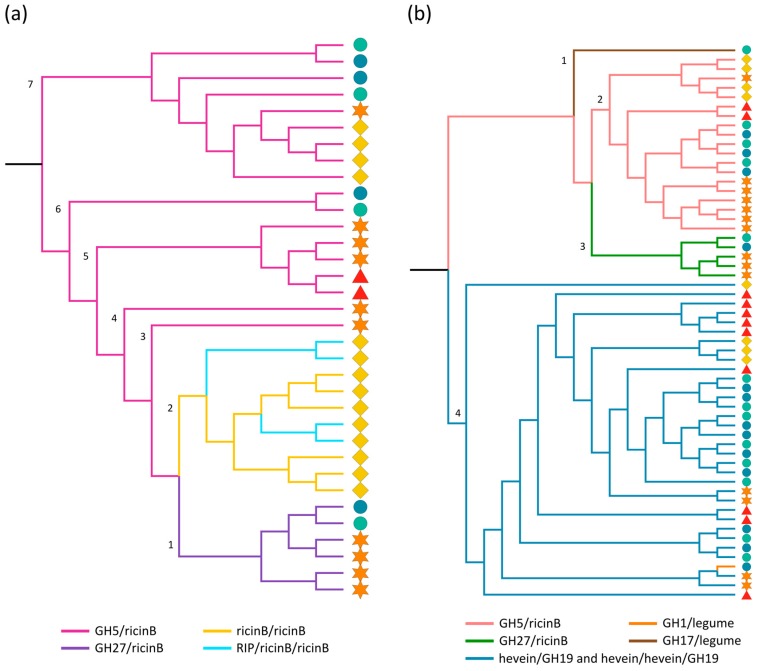
Midpoint rooted cladogram constructed with all ricin B (**a**) and GH (**b**) domain sequences from Arabidopsis (red triangle), soybean (orange star), cucumber (yellow rhomb) and rice (*japonica*: green circle, *indica*: blue circle). Numbers indicate the clade numbers and the colored branches correspond to the different domain architectures of the full-length ricin B (**a**) and GH (**b**) sequences.

In the genomes of Arabidopsis, soybean, cucumber and rice, the ricin B lectin domain is found to be part of multi-domain proteins; including the combination with a GH27 or a GH5 domain, a second ricin B domain, or in the RIP/ricin B/ricin B architecture. The maximum likelihood tree built using the protein sequences of all ricin B domains (Figure 1a), clearly reveals the closer relationship of ricin B domains that occur as part of the same domain architecture. Clade 1 groups together all ricin B lectin domains that are part of the GH27/ricin B domain architecture. The ricin B lectin domains from the cucumber specific ricin B/ricin B and RIP/ricin B/ricin B architectures (clade 2) are more closely related to each other than to those originating from other domain architectures, indicating that these domain combinations share a common ancestor as already suggested in other studies [63]. Clades 3–7 of the phylogenetic tree group together all the ricin B domains that are fused to a GH5 domain. Notably, within a certain clade, homologs of the same species often share the closest phylogenetic relationship.

Phylogenetic analyses of the GH domains (Figure 1b) support the findings from the ricin B tree. The GH5 and GH27 sequences are clustered in two separate clades (clade 2 and 3, respectively) of the phylogenetic tree, in a similar way to the corresponding ricin B domains in the ricin B tree. What is more, the subbranches are organized analogously (Figure S1), suggesting that the GH and ricin B domains evolved together and that the GH/ricin B domain combinations did not result from a rearrangement of the individual protein domains. Clade 4 contains the GH19 sequences and the unique GH1 sequence from *Oryza sativa* ssp. *indica*. Similar to the other clades, the subclades reflect the origin of the retrieved sequences. Surprisingly, the GH1 sequence shows a close connection to two GH19 domain sequences from soybean even though they are not part of the same clan of GHs (available online: www.cazy.org). A separate branch (clade 1) only containing the GH17 sequence from *Oryza sativa* ssp. *japonica* completes the phylogenetic tree. In this context, several studies investigated the evolution of glycoside hydrolase subfamilies. However, these reports mainly focused on different GH subfamilies, or were confined to bacterial and/or fungal species. Aspeborg and coworkers analyzed the large GH5 subfamily in plants, and group the GH5/ricin B proteins from Arabidopsis, rice and soybean identified in our study, together in a special cluster (GH5_11) similar to our phylogenetic analysis [64].

The ricin B domain refers to the lectin domain of ricin, a protein identified in *Ricinus communis* and known for its high toxicity to mammalian and other eukaryotic cells [65]. Ricin is a ribosome-inactivating protein (RIP) composed of an *N*-glycosidase domain (A chain or RIP domain) and a lectin domain (B chain or ricin B domain) [66]. Multi-domain proteins with this domain architecture are also referred to as type-2 RIPs. The lectin activity of the ricin B domain has been studied extensively and shows specificity towards galactose and *N*-acetylgalactosamine. The toxicity of ricin is mainly attributed to the *N*-glycosidase domain, though interaction of the lectin domain with galactosylated receptors on the cell surface helps the toxin to enter the cell [67]. The toxicity of some ricin homologs from *Abrus precatorius*, *Sambucus nigra* and *Viscum album* has been confirmed but it should be mentioned that not all proteins are as toxic as ricin [68]. Expression analysis and overexpression of type-2 RIPs provide evidence that these proteins play a role in plant defense of the plant against pathogens (tobacco mosaic virus) and pest insects (caterpillars, aphids) [69,70,71,72,73].

In cucumber, some ricin B lectins composed of two ricin B lectin domains are found. One of these proteins is referred to as XSP30 and was proposed to represent a signaling molecule produced in the xylem parenchyma and pericycle cells of cucumber roots and controlled by gibberellic acid [74]. Although the cucumber RIP/ricin B/ricin B homologs show high sequence similarity with the ricin sequence, their toxicity remains to be investigated [43].

Although GH/ricin B combinations have been identified in several of the species under study, this type of multi-domain protein has not yet been investigated in plants. In bacteria, combinations of such domains have been reported previously, and were shown to be involved in the degradation of insoluble or complex polysaccharides. These GH/ricin B proteins comprise an unusual combination of a carbohydrate-degrading (GH) and a carbohydrate-binding (lectin) domain and in bacterial homologs, the latter has been acknowledged to assist the enzymatic activity [75,76,77]. Both the GH5 and GH27 family are large families of glycoside hydrolases, grouping GHs with diverse enzymatic activities (available online: www.cazy.org) [64,78].

#### 2.3.2. Nictaba and F-Box Domains

**Figure 2:**
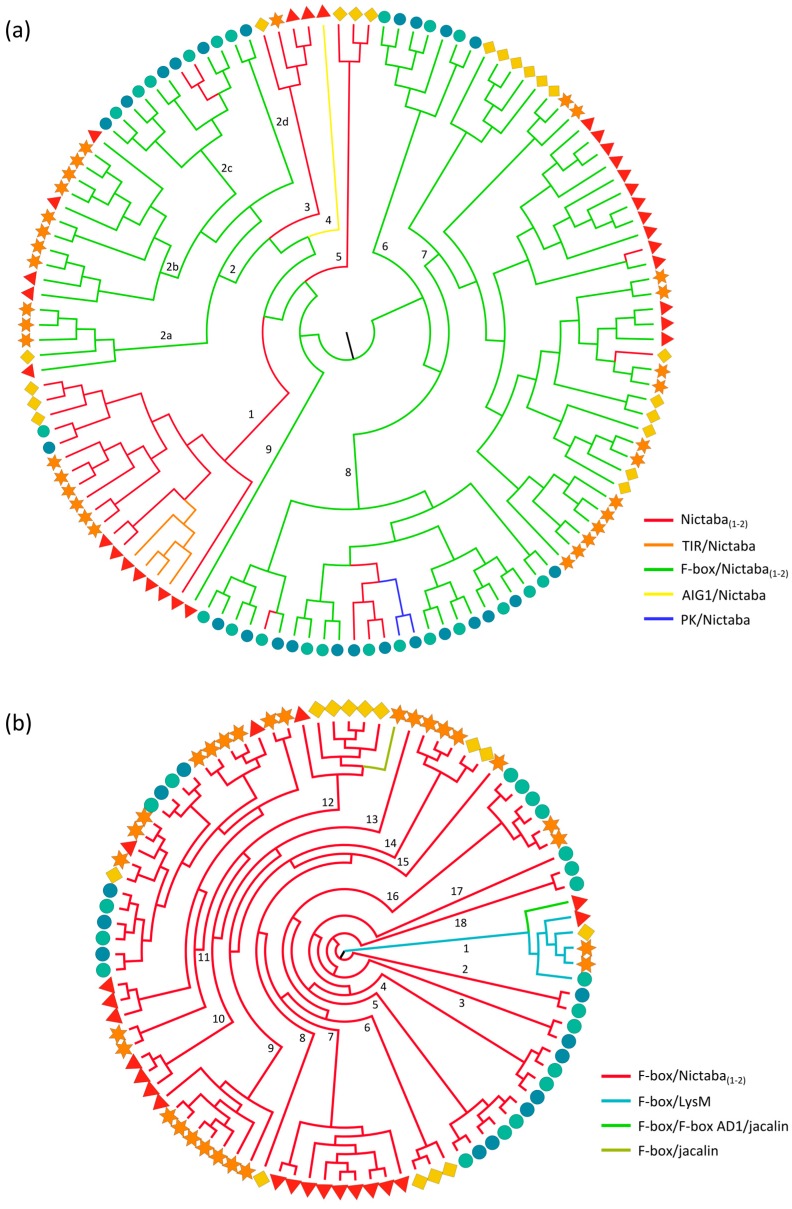
Evolutionary relationships of Nictaba (**a**) and F-box (**b**) sequences from Arabidopsis (red triangle), soybean (orange star), cucumber (yellow rhomb) and rice (*japonica*: green circle, *indica*: blue circle). The colored branches of the midpoint rooted maximum likelihood tree reflect different domain architectures of the full-length Nictaba (**a**) and F-box (**b**) sequences and the numbers specify the clade number.

Proteins containing the Nictaba domain or combinations of an F-box and a Nictaba domain are widespread in plants [30] and were identified in all species under study, with the F-box/Nictaba architecture being the most abundant. The phylogenetic tree built from the Nictaba domains (Figure 2a) reflects the domain organization of Nictaba-containing proteins. There are three clades (clade 1, 3 and 5) containing only sequences representing single-domain proteins. The two smallest clades 3 and 5 encompass exclusively dicot sequences, while the large clade 1 brings together sequences from both monocot and dicot origin. Within this clade, a smaller subclade groups together all Arabidopsis-specific sequences from the TIR/Nictaba domain architecture, implying that these all evolved from a recombination of the TIR and Nictaba in Arabidopsis, followed by multiple duplication events. The Nictaba domain sequence from the unique AIG1/Nictaba architecture is separated in clade 4 of the phylogenetic tree.

Clades 6–9 in the phylogenetic tree are mainly composed of Nictaba domains originating from F-box/Nictaba proteins. Within these clades, a few sequences correspond to single-domain proteins only containing the Nictaba domain, possibly due to loss of the F-box domain during evolution. These rearrangements are isolated events and did not always occur in the closest homologs, implying that they took place after the diversification of the different species and/or subspecies. One of the subclades of clade 8 also clusters the sequences derived from PK/Nictaba proteins, a protein domain combination that most probably evolved after the deletion of the F-box domain. Noteworthy, clade 2 with Nictaba sequences originating from the F-box/Nictaba proteins is more separated from the other clades (6–9) containing sequences with the same domain architecture. This clade can be further separated into four subclades: clades 2c and 2d only contain sequences from rice while clades 2a and 2b encompass different dicot sequences. More generally, this organization among subclades also applies to the other subclades of the phylogenetic tree, which also group sequences of the same origin.

Overall, there is a clear separation between the Nictaba domain sequences originating from Nictaba and F-box/Nictaba proteins, indicating that the F-box/Nictaba protein domain combination originated early in a shared ancestor of plants, long before the divergence of monocot and dicot plants. These conclusions are in correspondence with a previous study, in which Nictaba homologs from 15 plant genomes were analyzed [30].

For proteins composed of two Nictaba domains, the individual Nictaba domain sequences are organized in the same (soybean) or closely related (*Oryza sativa*) clades of the cladogram (Figure S2). This implies that the second domain originated from an in tandem duplication of the original *Nictaba* domain. For rice, these *F-box/Nictaba/Nictaba* genes were also later duplicated in the genome. The number of Nictaba homologs in *Oryza sativa* ssp. *japonica* and *indica* is highly comparable since orthologs for each particular sequence always group together in the same subclades of the phylogenetic tree (Figure S2).

For comparison, a phylogenetic tree was also constructed using all F-box domain sequences from the putative lectin proteins (Figure 2b and Figure S3). The tree shows more than 15 smaller clades, each mostly containing domain sequence orthologs from one species. Only a small number of clades group together sequences from different species. Clades 1 and 11 contain both monocot and dicot species, one of them (clade 1) brings together sequences from the F-box/F-box AD1/jacalin and F-box/LysM architecture. 

Surprisingly, the F-box/F-box AD1/jacalin sequence from Arabidopsis and the F-box/jacalin sequence from soybean (clade 12) are in two distinct clades of the phylogenetic tree, suggesting that they are the result of two independent domain fusion events which could also explain why these sequences are not found in the other species since they do not share a common ancestor in land plants. The F-box sequences from the F-box/LysM and F-box/F-box AD1/jacalin sequences from Arabidopsis and rice also clustered in distinct clades in studies including all F-box sequences present in these species, supporting our results [79,80].

Remarkably, the corresponding F-box domains of the Nictaba domain sequences from clade 2 in Figure 2a are also found in a distinct clade (11) of the phylogenetic tree based on the F-box domain sequences (Figure 2b). This again supports the idea that the F-box and Nictaba domain evolved together, and have diverged from an ancestral protein, as suggested in a previous study [30].

Nictaba was originally discovered in tobacco leaves treated with methyl jasmonate [81]. Later, different jasmonate derivatives as well as insect herbivory were reported to specifically enhance the expression of this gene [82,83]. Furthermore, Nictaba as well as two Nictaba homologs from soybean exert insecticidal activity. Moreover, Arabidopsis overexpression lines for the Nictaba homologs from soybean also showed reduced disease symptoms upon *Pseudomonas syringae* infection [84]. Similarly, overexpression of *PP2-A1*, an Arabidopsis homolog, repressed phloem feeding of *Myzus persicae* and addition of the protein to an artificial diet significantly reduced the weight gain of aphids [85,86]. It is hypothesized that within plant cells, Nictaba acts as a stress signaling molecule through interaction with *O*-GlcNAc-modified histones [87].

In Arabidopsis, some Nictaba domains are found in combination with the TIR or AIG1 (avirulence induced gene 1) G-type domain, which are known for their roles in pathogen detection and defense responses [88,89], again supporting a role of Nictaba proteins in plant defense. However, F-box/Nictaba protein combinations are the most abundant of all Nictaba homologs. The F-box domain is a component of the SCF (Skp, Cullin, F-box) complex, which functions in the proteasome-mediated protein degradation [90,91,92,93]. The combination of this protein domain with the carbohydrate-binding Nictaba domain could potentially facilitate degradation of glycoproteins in plants [79,94]. Expression analysis revealed that some *F-box/Nictaba* genes from Arabidopsis and rice are stress inducible [92,95,96]. Overexpression lines for two F-box/Nictaba-related genes *PP2-B10* and *PP2-B11* from Arabidopsis showed a higher tolerance to *Pseudomonas syringae* infections and high salinity, respectively [97,98], confirming the link between Nictaba homologs and plant stress responses.

#### 2.3.3. Legume Lectin Homologs

**Figure 3:**
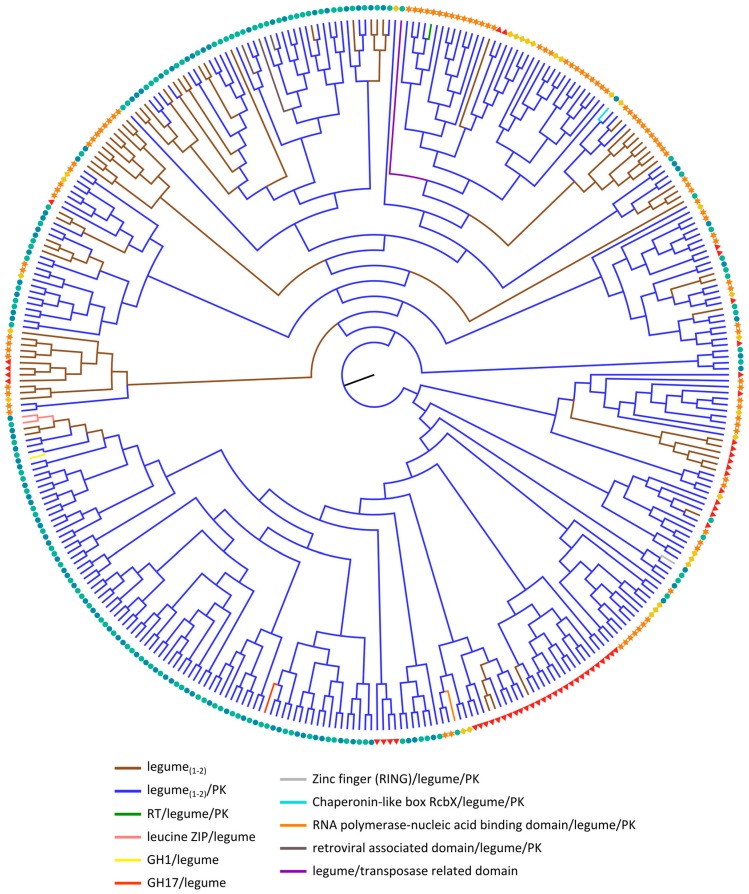
Midpoint rooted cladogram constructed with all legume lectin domain sequences from Arabidopsis (red triangle), soybean (orange star), cucumber (yellow rhomb) and rice (*japonica*: green circle, *indica*: blue circle). The colored branches correspond to the different domain architectures of the full-length legume lectin sequences.

Phylogenetic analysis of the legume lectin domain sequences (Figure 3) indicates that except for some small clades (containing only 1–11 sequences), none of the other clades group together legume lectin domains originating from the same domain architecture. What is more, the results demonstrate a lineage-wise organization, followed by domain rearrangements within the different lineages. This observation is inconsistent with the phylogenetic analyses of ricin B and Nictaba domains (Figure 1a and Figure 2a), in which the tree was predominantly based on the domain arrangement of the corresponding proteins, underlining that the individual lectin domains have undergone dynamic evolutionary processes. As a result, the different legume lectin domains from a particular species that share a similar domain organization are scattered over distinct branches of the phylogenetic tree. Legume lectin sequences from different organisms are found in some clades, but the closest homologs of Arabidopsis, soybean, cucumber and rice are each clustered together respectively, signifying that the expansion of the legume lectin sequences has occurred after the divergence of the different species. Surprisingly, the phylogenetic tree built using only the PK domain sequences that were found in combination with lectin domains, clusters all PK domains that are part of legume lectin homologs (Figure S5), except for one rice sequence. Previous reports, focusing on these legume-type receptor kinases in Arabidopsis proposed a classification system based on their phylogenetic analyses of the legume lectin, PK and full-length sequences [27,99]. This classification remains consistent with our data, even though our analysis includes four additional genomes (soybean, cucumber, *Oryza sativa* ssp. *japonica* and *Oryza sativa* ssp. *indica*) (Figure S4). 

Legume lectins were originally discovered in leguminous plants. Though legume lectins have been considered as a group of lectins that were restricted to leguminous plants for many decades, compelling evidence now shows that the so-called legume lectin domain is also widespread in the plant kingdom. Legume receptor-like kinases (RLKs) or L-type RLKs are a specific group of legume lectin homologs with diverse functions. Functional characterization of many L-type RLKs reported on the involvement of this type of protein in symbiosis [100,101,102], defense mechanisms against *Phytophthora* ssp. [25,103,104,105,106,107], *Botrytis cinerea* [108], enhanced tolerance against insects [109], bacterial infection [110,111], salinity [108,112] and stomatal closure [111] in multiple plants. Recently, DORN1 encoded by the *AtLecRK-I.9* gene was identified as the first receptor for extracellular ATP [113] and recognition of extracellular ATP by this plasma membrane localized L-type RLK induces the innate immune system [114].

#### 2.3.4. GNA Homologs

**Figure 4:**
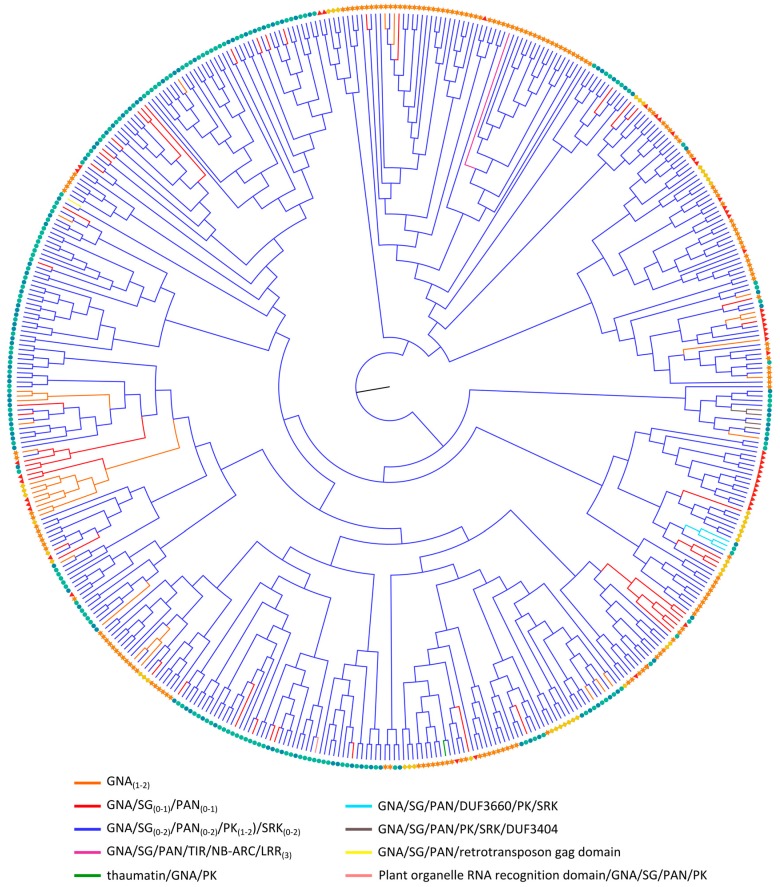
Evolutionary relationships of GNA sequences from Arabidopsis (red triangle), soybean (orange star), cucumber (yellow rhomb) and rice (*japonica*: green circle, *indica*: blue circle). The colored branches of the midpoint rooted maximum likelihood tree reflect different domain architectures of the full-length sequences.

Analysis of the evolutionary relationships of the GNA homologs (Figure 4 and Figure S6) shows resemblance to the data obtained for the legume lectins (Figure 3). The GNA sequences are generally not clustered according to the domain architecture, nor based on the origin of the species from which they were identified. Most of the domain architectures of the GNA proteins are conserved between Arabidopsis, soybean, cucumber and rice, indicating that these multi-domain configurations were established before the divergence of these plant species. Nevertheless, some novel domain arrangements were identified that are mostly specific to rice. The GNA domains from soybean and rice protein sequences make up the largest group of the phylogenetic tree, which can be explained by the significant contribution of tandem and segmental duplication in the expansion of the GNA family in soybean [37]. Intriguingly, the expansion of this family in *Oryza sativa* ssp. *indica* is less pronounced compared to *Oryza sativa* ssp. *japonica*. This is probably the consequence of differential expansion among the two subspecies.

All clades contain GNA domain sequences from proteins with the GNA/SG/PAN/PK/SRK domain organization in combination with GNA sequences from other protein configurations. However, the latter, i.e., GNA domains from single-domain proteins, cluster mostly in separate subbranches. The presence of GNA single-domain proteins in clades that are predominantly composed of GNA domains from the GNA/SG/PAN/PK/SRK combinations can be explained by recent single-step events in which the PK domain and/or other non-lectin domains were lost. Thus, evidence is accumulating for a large GNA/SG/PAN/PK/SRK family in the common ancestor of flowering plants. Subsequently, this family has further expanded and diverged in the different lineages, resulting in diverse family sizes (Table 1). Notably, most subfamilies are specified by lineage-specific expansions and rearrangements.

Reports on the evolution of the GNA family in literature are limited. One study dealing with GNA RLKs in Brassicaceae concluded that some gene fusion events in the common ancestor of land plants resulted in almost all GNA RLKs, which is in agreement with our hypothesis [33]. Similar assumptions were made in a comparative analysis of protein sequences containing PK domains from Arabidopsis and rice [115]. In this context, Figure S5 again shows that the PK domains from GNA homologs are grouped together in separated branches of the phylogenetic tree. The PK domains present in different lectin sequences are clearly lectin-specific and were not recombined between different lectin domains during evolution.

GNA or G-type RLKs are best studied in Brassicaceae and also known as S-domain RLKs due to the presence of the S-locus glycoprotein domain which is involved in pollen self-incompatibility [33,116]. Other G-type RLKs were demonstrated to function in plant defense against both biotic and abiotic stress [24,26,117,118,119]. So far, there are no reports on the functional analysis of homologs in Arabidopsis, soybean or cucumber. Yet, several genes were shown to be upregulated in plants exposed to diverse stresses [120,121] and one Arabidopsis GNA gene is co-expressed with *BIR2*, which plays a role in plant innate immunity [122]. OsSIK2, a rice GNA domain containing protein is induced by a broad range of abiotic stresses and overexpression of the gene confers salt and drought tolerance [123]. Another rice protein belonging to the G-type RLK family, OslecRK, is also involved in the plant innate immunity and promotes seed germination [124]. A gene cluster of rice GNA RLKs (*OsLecRK1*-*OsLecRK3*) was shown to confer broad-spectrum and durable insect resistance [125]. In a study by Chen and coworkers, transgenic plants carrying a different member of GNA RLKs conferred resistance to *Magnaporthe grisea* [126].

Other GNA domain architectures including the thaumatin, LRR and NB-ARC domain suggest that these proteins are stress-related given the involvement of these domains in disease resistance and stress adaptation [31,127,128,129,130].

#### 2.3.5. Jacalin-Related Lectin Domains

**Figure 5:**
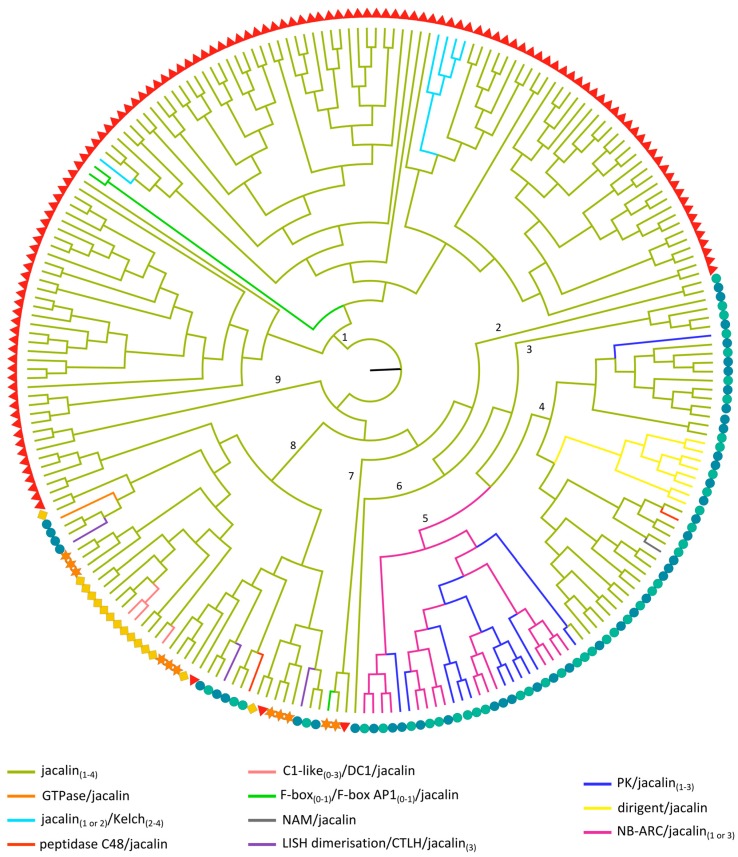
Evolutionary relationships of JRL sequences from Arabidopsis (red triangle), soybean (orange star), cucumber (yellow rhomb) and rice (*japonica*: green circle, *indica*: blue circle). The colored branches of the midpoint rooted maximum likelihood tree reflect different domain architectures of the full-length JRL sequences and the numbers specify the clade number.

Analysis of the phylogenetic relationships between the different jacalin domains reveals that lectin sequences derived from one species show a closer relationship (Figure 5). This is in contrast with the phylogenetic relationships of some other lectin domains (Figure 1a and Figure 2a) where clustering based on the domain architecture was observed. Within the phylogenetic tree, nine clusters can be distinguished. The majority of the jacalin homologs from Arabidopsis group into two separate clusters of the phylogenetic tree (cluster 1 and 9, respectively). Likewise, most jacalin sequences from rice are clustered in clades 2–6. Clade 8 contains sequences of all species. Within this clade, there are several subclades, grouping all the jacalin sequences from cucumber including a subclade with the sequences from the C1-like/divergent C1 (DC1)/jacalin domain architecture. Surprisingly, the three jacalin domains from the Lis homology dimerization domain (LISH dimerization)/CT11-RanBPM C-terminal to LISH (CTLH)/jacalin domain combination are found in different subclades of clade 8, suggesting that these domain repeats do not result from in tandem duplications. The jacalin domain from the GTPase/jacalin domain combination is also part of clade 8 and possibly originates from the fusion of the GTPase domain to an already existing jacalin in rice.

In agreement with the phylogenetic analysis of the F-box protein domains (discussed in 2.3.2), the jacalin domains of the (F-box/)F-box AD1/jacalin proteins from Arabidopsis and the F-box/jacalin protein from soybean cluster in two different branches (clade 1 and 7, respectively) of the phylogenetic tree. This observation again confirms that these proteins do not share a common history.

In clades 2, 3, 6 and 9; only jacalin domains from the single-domain architecture are grouped (Figure S7). All other clades contain jacalin sequences from both the single-domain architecture and multi-domain architectures involving only jacalin domains and multi-domain proteins with additional non-lectin domains. Interestingly, in clade 5, only one jacalin domain from a single domain architecture is found. All other sequences in this clade originate from NB-ARC/jacalin or PK/jacalin architectures, suggesting that the additional non-lectin domain was lost in the earlier described sequence. The close connection between the jacalin domains from the NB-ARC/jacalin and PK/jacalin arrangements indicates that these proteins share a common ancestor. Remarkably, the third jacalin domain from one of the PK/jacalin/jacalin/jacalin protein combinations is grouped in clade 4, signifying a unique and more recent rearrangement. This is not in full agreement with the phylogenetic tree built with all PK domain sequences that are part of lectin architectures (Figure S5) in which all PK sequences from jacalin-containing proteins grouped together.

The dirigent/jacalin proteins are restricted to rice and most probably evolved from a recombination of the dirigent and jacalin domain before the diversification of rice. This event was followed by multiple tandem duplication events, resulting in the presence of these jacalin domains in a separate branch of clade 4.

Clade 4 also contains the jacalin domain from the exclusive no apical meristem (NAM)/jacalin protein and one of the peptidase C48/jacalin-related jacalin domains. The other jacalin domain originating from the same domain architecture is grouped in clade 8, suggesting that the recombination of the jacalin domain with the peptidase C48 domain occurred twice, independently of each other.

JRLs can be classified into 2 subgroups: the vacuolar galactose-binding JRLs and the nucleocytoplasmic mannose-binding JRLs [131]. While mannose-specific lectins are widespread in Viridiplantae, the galactose-specific JRL are mainly confined to the Moraceae family. In this study, a small number of JRLs containing a signal sequence were identified in Arabidopsis (3 sequences) and rice (1 sequence). In soybean and cucumber, none of the sequences contains a signal peptide. The sequences from Arabidopsis are composed of two in tandem arrayed jacalin domains and the first and second domain of these sequences cluster closely together in the phylogenetic tree (Figure S7). The same observations were made based on the phylogenetic tree of Arabidopsis JRLs by Eggermont and co-workers [39]. 

Several JRLs are involved in disease resistance, abiotic stress signaling, wounding and plant defense. This is reflected in the domain architecture of these lectins, where the jacalin domain is fused to domains related to stress response and defense (e.g., dirigent or disease-response domain, leucine rich repeats (LRR), NB-ARC). The combination of a jacalin and dirigent domains has only been reported in Poaceae species [132] and these chimeric lectins were shown to be involved in plant defense [34]. In addition to a role in plant defense, Ver2, a dirigent-JRL from wheat, was shown to be associated with vernalization-induced *O*-GlcNAc signaling and intracellular motility [133].

Next to the chimeric JRLs, also the JRLs solely composed of jacalin domains were reported as stress-related proteins. Transcript levels for the rice gene *Orysata* are upregulated in response to several stresses (salt stress, senescence, insect infestation and *Magnaporthe grisea* infection) and hormonal treatments (jasmonic acid and abscisic acid) [134,135,136,137,138]. Recently, this gene was identified in Saltol-1, a major quantitative trait loci related to salt stress [139]. Overexpression of *Orysata*, also known as SALT, in rice plants improves salinity tolerance [140] and enhances resistance to *Magnaporthe grisea* infection [141]. The Arabidopsis-related JAX1 was shown to confer broad but specific resistance to potex viruses [142]. Similarly, the *Arabidopsis* JAC1 homolog prevents systemic infection of the tobacco etch potyvirus, the plum pox virus, and the lettuce mosaic virus [143,144,145,146]. The NB-ARC domain is a novel signaling motif found in bacteria and eukaryotes, and is shared between plant resistance gene products and regulators of cell death in animals [147].

Other domains associated with the jacalin domain are annotated to be related to signal transduction. The C1-like and DC1 domain found in cucumber can bind to diacylglycerol [148], a membrane bound lipid secondary messenger. In rice, multi-domain proteins consisting of a jacalin domain with the GTPase domain and GTPase binding domains (LISH and CTLH) were identified. GTPases function as molecular switches in several processes and signaling cascades [149]. However, the precise role of these multi-domain proteins still needs to be elucidated.

#### 2.3.6. LysM Domains

**Figure 6:**
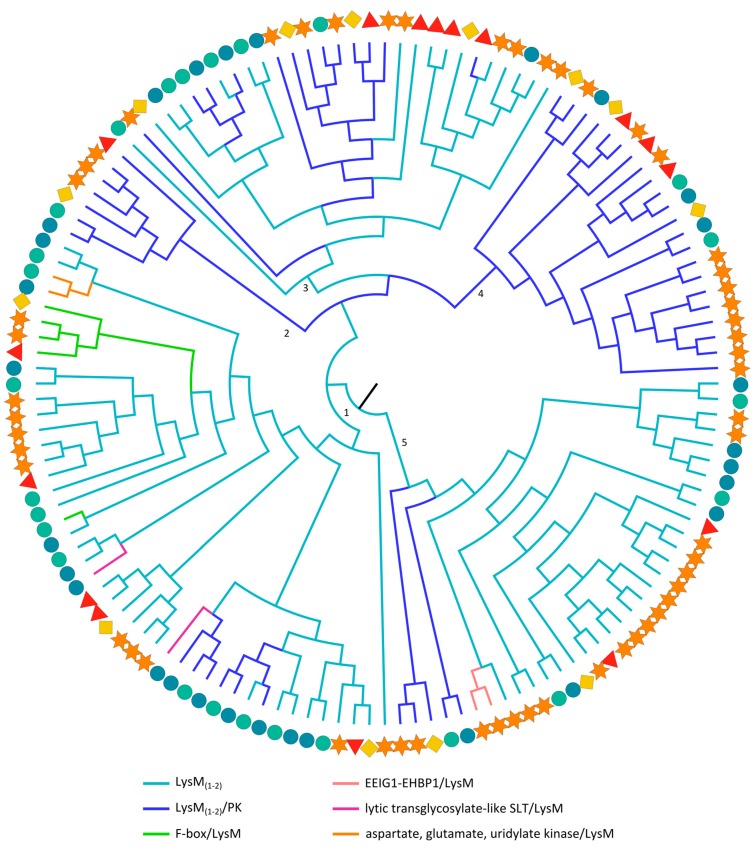
Evolutionary relationships of LysM sequences from Arabidopsis (red triangle), soybean (orange star), cucumber (yellow rhomb) and rice (*japonica*: green circle, *indica*: blue circle). The colored branches of the midpoint rooted maximum likelihood tree reflect different domain architectures of the full-length sequences.

The maximum likelihood tree with the LysM domain sequences (Figure 6) reveals some clustering based on the architecture of the corresponding LysM lectin sequences. However, not all clusters with the same architecture are grouped in one branch, suggesting that the different architectures arose several times during evolution. Clade 2 and 4, for example, only group LysM domains from the LysM/PK multi-domain architecture, but LysM domain sequences from this protein architecture are also found in all other clades. Clade 1 is the most diverse, containing LysM domains from five different domain architectures. The LysM domains that originate from homologous domain architectures are generally more closely related. However, the LysM domains from the two rice-specific lytic transglycosylase-like soluble lytic transglycosylase (SLT)/LysM proteins are in two different subbranches. The same occurs for the F-box/LysM-related LysM domains: the rice LysM domain from this architecture is found in a distinct subclade while all dicot sequences group together (clade 1). This might be due to further diversification of the rice LysM domain after the split of the monocots and dicots. Next to the LysM and LysM/PK sequences, clade 5 also groups the two EEIG1-EHBP1/LysM-related LysM domain sequences from soybean. 

All clades contain LysM domains from all different species, but lineage-specific subclades reveal the expansion through duplication events. Surprisingly, for proteins with two in tandem arrayed LysM domains, the two LysM domains are not clustered in the same clade (Figure S8). While the second LysM domain sequences are grouped in clades 1 and 4, the LysM domains corresponding to domain one are part of clade 3. These observations are in agreement with an earlier study on Arabidopsis LysM lectins [39]. The diversity of LysM motifs was previously reported in the evolutionary studies by Zhang et al. [35,150]. Although these studies focused on the evolution within the different LysM types across all kingdoms, our study focuses on the evolution of the whole LysM family in some core angiosperms.

LysM RLKs are cell wall localized receptors that recognize GlcNAc moieties in various types of bacterial peptidoglycans, fungal chitins and rhizobacterial Nod factors. Some LysM RLKs play a role in plant defense as they are part of the plant innate immunity. Next to the classical LRR receptor kinases, LysM RLKs are now acknowledged as true pattern recognition receptors and are involved in the perception of microbial signals [151,152]. GlcNAc-based elicitors such as lipochitooligosaccharides, chitin and peptidoglycan are perceived by a complex of LysM RLKs. In Arabidopsis, peptidoglycan is recognized by a protein complex including CERK1 (chitin elicitor receptor kinase), a LysM protein with functional intracellular PK domain; and LYM1 or LYM3, LysM homologs that are anchored to the plasma membrane by glycosylphosphatidylinositol (GPI). Analogously, chitin is perceived by CERK1 and LYK5, the latter being a LysM homolog with a dysfunctional kinase domain. The chitin receptor in rice is slightly different since CERK1 associates with CEBiP (chitin elicitor binding protein), a GPI anchored LysM protein. These LysM RLKs are the only lectin pattern recognition receptors for which it has been unambiguously shown that they depend on carbohydrate-lectin interaction for their biological activity [153,154].

Next to their role in plant innate immunity, some LysM RLKs are involved in symbiosis. In legumes and other plants, symbiosis signaling involves recognition of the beneficial microorganisms such as rhizobacteria and mycorrhiza [152,155,156]. The receptor complexes of LysM RLKs that perceive the elicitors have been identified in several legumes (*Lotus japonica*: LjNFR1/LjNRF5, *Glycine max*: GmNRF1/GmNFR5, *Medicago truncatula*: LYK3/NFP9) [155,157,158,159,160,161].

#### 2.3.7. Hevein Domains

**Figure 7:**
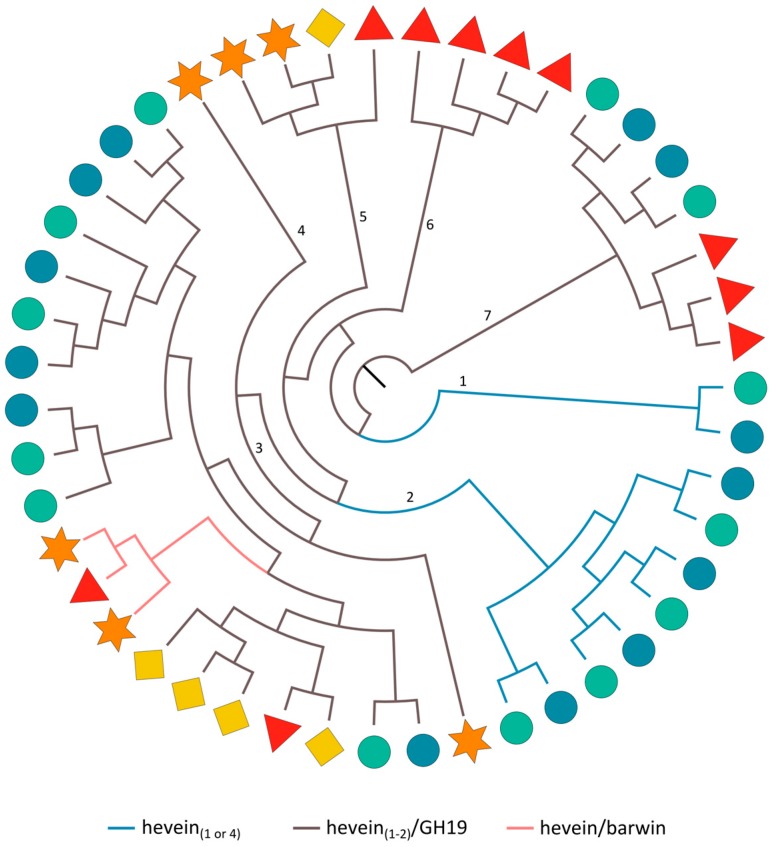
Midpoint rooted cladogram constructed with all hevein domain sequences from Arabidopsis (red triangle), soybean (orange star), cucumber (yellow rhomb) and rice (*japonica*: green circle, *indica*: blue circle). Numbers indicate the clade numbers and the colored branches correspond to the different domain architectures of the full-length hevein sequences.

The maximum likelihood tree made using the protein sequences of the hevein domains (Figure 7), uncovers the close phylogenetic relationship of hevein domains that are part of sequences with the same domain arrangement. Clade 1 and 2 group all rice-specific hevein domains from sequences comprising a single or four in tandem arrayed hevein domains, respectively. The hevein domains from the hevein/barwin combination are present in a subclade of clade 3 while the other subclades of clade 3 and clades 4–7 group all hevein domains from the hevein/GH19 or hevein/hevein/GH19 protein architecture. Thus, the hevein/barwin protein most probably originated from a hevein/GH19 protein in which the GH19 domain was exchanged with a barwin domain. The hevein/barwin domain combination is present in many different plant species [16], indicating that this protein rearrangement took place in a common ancestor of land plants and these genes were apparently retained in most lineages. Figure 7 undeniably illustrates that within a certain clade, homologs of a certain species often share the closest phylogenetic relationship. For the *Oryza sativa* ssp. *japonica* and *indica* sequences containing four hevein domains (clade 2), the corresponding hevein domains from both subspecies are clustered together (Figure S9). This is also the case for the two hevein domains from the cucumber hevein/hevein/GH19 sequence that are part of clade 3. For the hevein/GH19 protein sequences, clustering of the GH19 domain sequences (Figure 1b) resembles the organization of subclades in the phylogenetic tree made with the hevein sequences (Figure 7).

Hevein/barwin domain combinations are also known as class I pathogenesis-related (PR) 4 proteins [162]. The barwin domain was originally identified in barley, and plays a role in the defense response against fungal infection [163]. Class I PR 4 genes are generally upregulated upon pathogen attack, but other stress factors can also regulate PR 4 transcript levels. In Arabidopsis, the expression of the class I PR 4 gene is upregulated upon ethylene, methyl jasmonate, abscisic acid, high salt treatment and *Alternaria brassicicola* infection [164,165,166,167,168]. Given the chitin-binding properties of the hevein domain, it is thought that PR 4 proteins contribute to plant defense against fungi [169].

Class I and class IV chitinases consist an N-terminal hevein domain and a C-terminal GH19 catalytic domain [78]. *AtEP3*, an Arabidopsis homolog of this family, is also considered as a stress-responsive gene since its expression can be upregulated upon *Xanthomonas campestris* infection, UV light, SA treatment and wounding [170,171]. Analogously, transcript levels of a soybean class I chitinase were also increased in response to *Phytophthora sojae* infection [172]. RNA-seq analysis demonstrated that two rice class I chitinase genes (*CHIT17* and *CHIT7*) were significantly upregulated in a *Magnaporthe oryzae* resistant rice cultivar [173]. Transgenic rice plants that overexpress the rice *Cht-2* or *Cht-3* chitinase gene showed a higher resistance against *Magnaporthe grisea* [174]. Moreover, the expression of rice class I chitinases (*rcc2* or *rcg3*) in banana enhanced resistance towards black leaf streak disease [175] and the introduction of another rice homolog (*RC24*) in wheat helped the plant to cope with strip rust infection [176].

## 3. Materials and Methods

### 3.1. Identification and Classification of Lectin Genes

Putative lectin genes from Arabidopsis, soybean, cucumber and rice were identified using a combination of BLAST, InterProScan5 and hidden Markov models. Protein sequences encoding a representative protein from each lectin family (for more details, see [37]) were used as a query for BLASTp searches (E-threshold: 10, comparison matrix: BLOSUM62, word length: 3). BLASTp was performed with the Arabidopsis (TAIR annotation release 10) and soybean (Wm82.a2.v1) datasets available from Phytozome v. 10 (available online: https://phytozome.jgi.doe.gov/pz/portal.html). The Cucurbit Genomics Database (available online: http://www.icugi.org/cgi-bin/ICuGI/index.cgi) and MSU Rice Genome Annotation Project Database (RGAP release 7) [177] and Phytozome v. 10 were used to perform BLASTp searches for cucumber and rice, respectively. Lectin genes in the genome of *Oryza sativa* ssp. *indica* (ASM465v1) were identified using Ensembl Plants (available online: http://plants.ensembl.org/index.html). Subsequently, the top hit was used for a second BLASTp search and all retrieved sequences were selected as candidate lectin genes. The corresponding protein sequences were downloaded and the presence of the lectin domains and any other annotated protein domains was identified using InterProScan5 (available online: http://www.ebi.ac.uk/Tools/webservices/services/pfa/iprscan5_soap) with default settings [178]. Protein sequences that lacked conserved lectin domains were removed from the dataset. The presence of signal peptides and transmembrane domains was analyzed with the SignalP 4.1 Server [179] and the TMHMM Server v. 2.0 [180], respectively.

### 3.2. Phylogenetics

Maximum likelihood trees were generated using the amino acid sequences of the protein domains. For protein sequences containing multiple domains of the same type, the domains were separated and all domain sequences were included in the analysis. The domain sequences were aligned with MUSCLE using default settings [181]. The alignment was performed online (available online: http://www.ebi.ac.uk/Tools/msa/muscle/) or locally using molecular evolutionary genetics analysis 7 (MEGA7) [182]. Subsequently, the aligned sequences were trimmed to generate blocks of conserved aligned sequences using the automated1 option of trimAl v. 3 [183]. Based on the trimmed alignments, maximum likelihood trees were built with RAxML v. 8.2.4 [184]. RAxML used the general time reversible gamma (GTRGAMMA) model with automated determination of the best amino acid substitution model, random number seed and distinct starting trees. Bootstrap iterations to assess the robustness of the generated trees were decided automatically. Phylogenetic trees were visualized and edited with FigTree v. 1.4.2 (available online: http://tree.bio.ed.ac.uk/software/figtree/).

### 3.3. Segmental and Tandem Duplications

Gene expansion through segmental and/or tandem duplications was analyzed with PLAZA [58].

## 4. Conclusions

In this study we presented an inventory of the lectin sequences in five genomes of model plants, including *Arabidopsis thaliana*, *Glycine max*, *Cucumis sativus*, *Oryza sativa* ssp. *japonica* and *Oryza sativa* ssp. *indica*. Our data demonstrated that most plant lectin families are present across all surveyed genomes. Although these findings are consistent with previous analyses made in dicot genomes, we are not aware of genome wide studies investigating the lectin sequences from monocot species. Furthermore, PLAZA analysis confirmed that all plant lectins under study are also present in *Amborella trichopodia* and *Physcomitrella patens*, signifying ancient lectin domain integrations and pointing out the occurrence and importance of lectins throughout the whole plant kingdom. 

Additionally, we performed a comparative and evolutionary genomics study of some particular plant lectin domains and some other protein domains that are found in combination with the lectin domains. A detailed analysis of the lectin sequences revealed a wide range of domain architectures, involving the fusions of lectin domains with other annotated protein domains. Parts of these reccurring additional protein domains have been reported to be involved in plant defense, signaling and/or development, which agrees with the idea that lectin domains play a role in plant growth and defense. Some of the multi-domain proteins are widespread, indicating the retention of ancient fusions in a common ancestor. In contrast, the many species-specific domain arrangements can be explained by lineage-specific retention of a certain domain rearrangement or by a recent event of protein domain fusion or domain loss in a particular species [12].

For some lectin families, there is significant variation in the number of lectin genes across flowering plants. Phylogenetics confirmed that duplication events and speciation are the driving source for this diversification. This is also reflected in the organization of lectin sequences in the phylogenetic trees. The JRL family clearly demonstrates a lineage-specific evolution while the ricin B family experienced extension of the different domain architectures in most species. These findings support the dynamic evolution of the different plant lectin families in respect to the domain organization and duplication events. In the future, it will be important to continue studying lectin families across species to enrich our understanding of their evolutionary history, but this method also has some limitations. The proteins encoded by the lectin genes are part of a larger and complex interactome, and it will be difficult to relate the emergence of a new protein domain combination to existing pathways. Therefore, a combined analysis of these traditional approaches in combination with co-expression networks would be advantageous and could expand our knowledge since recent studies demonstrated that co-expression networks are conserved also across species [185,186]. At the same time, we cannot exclude the presence of minor mistakes in our dataset due to missing annotation of genes or unidentified protein domains. 

It is clear that plants have a large variety of plant lectins at their disposal. During evolution, new lectin domain combinations have been created and used to the benefit of the plant to allow rapid adaptation to changing environmental conditions. Although the functionality of the putative lectin domains needs to be confirmed by experimental evidence, these lectins will most probably exert complementary activities since they are present in different subcellular/tissue specific locations and recognize different carbohydrate structures, either present in the plant or part of pathogens. Further functional investigation of these lectins will substantially contribute to our knowledge of the lectin complement and their importance for the plant.

## Figures and Tables

**Table 1:** Distribution of lectin genes in Arabidopsis, soybean, cucumber and rice.

Lectin Family	Predicted Genes									
	*Arabidopsis Thaliana*		*Glycine Max*		*Cucumis Sativus*		*Oryza Sativa* (*Japonica*)		*Oryza Sativa* (*Indica*)	
	#	%	#	%	#	%	#	%	#	%
Amaranthin	0	0.0	0	0.0	16	11.0	0	0.0	0	0.0
CRA	9	4.2	6	1.6	4	2.7	2	0.6	4	1.5
EUL	1	0.5	3	0.8	1	0.7	5	1.5	5	1.8
GNA	49	22.7	166	45.1	45	30.8	134	41.2	94	34.3
Hevein	10	4.6	6	1.6	4	2.7	10	3.1	10	3.6
Jacalin	50	23.1	5	1.4	8	5.5	30	9.2	33	12.0
Legume lectin	54	25.0	94	25.5	29	19.9	104	32.0	84	30.7
LysM	12	5.6	47	12.8	10	6.8	20	6.2	22	8.0
Nictaba	29	13.4	31	8.4	20	13.7	20	6.2	22	8.0
Ricin B	2	0.9	10	2.7	9	6.2	4	1.2	4	1.4
Total number of lectin sequences	216		368		146		329		278	

**Table 2:** Most prevalent domain architectures in *Arabidopsis thaliana* (*At*), *Glycine max* (*Gm*), *Cucumis sativus* (*Cs*) and *Oryza sativa* ssp. *japonica* (*Os ja*) and *indica* (*Os in*)^1^. CID: chitinase insertion domain, SG: S-locus glycoprotein, PK: protein kinase, SRK: S-locus receptor kinase, GH: glycoside hydrolase, F-box AD1: F-box associated domain type 1, NB-ARC: nucleotide binding domain shared by Apaf-1, R proteins, and CED-4; TIR: Toll/Interleukin-1 receptor; RIP: ribosome-inactivating protein domain.

Domain Architecture	*At*	*Gm*	*Cs*	*Os ja*	*Os in*	Schematic Representation
Amaranthin/amaranthin/aerolysin	0	0	16	0	0	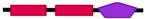
CRA	2	5	4	2	3	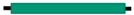
CRA/CID	7	0	0	0	0	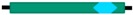
EUL_(1–2)_*	1	3	1	5	5	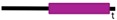
GNA	6	9	3	2	11	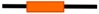
GNA/SG_(0-2)_/PAN_(0-2)_/PK _(0–2)_/SRK _(0–2)_	43	155	42	125	76	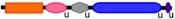
Hevein_(1 or 4)_	0	0	0	2	2	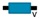
Hevein/barwin	1	2	0	0	0	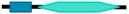
Hevein_(1–2)_/GH19	9	4	4	8	8	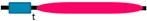
Jacalin_(1–4)_	44	4	5	17	21	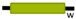
F-box_(0–1)_/F-box AD1_(0–1)_/jacalin	2	1	0	0	0	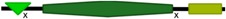
Jacalin_(1–2)_/Kelch1_(1,2 or 4)_	4	0	0	0	0	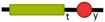
C1-like_(1–3)_/DC1/jacalin	0	0	3	0	0	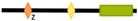
PK/jacalin_(1–3)_	0	0	0	4	1	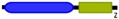
Dirigent/jacalin	0	0	0	4	4	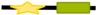
NB-ARC/jacalin_(1–3)_	0	0	0	3	4	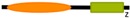
Legume_(1–2)_	13	27	2	18	12	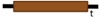
Legume_(1–2)_/PK	41	66	27	79	68	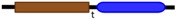
LysM_(1–2)_	6	25	3	12	11	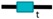
F-box/LysM	1	2	1	1	0	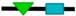
LysM_(1–2)_/PK	5	18	6	7	10	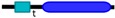
Nictaba_(1–2)_	7	6	8	4	4	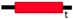
F-box/Nictaba_(1–2)_	18	25	12	15	16	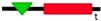
TIR/Nictaba	4	0	0	0	0	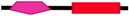
GH5/ricin B	2	6	4	3	3	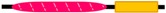
GH27/ricin B	0	4	0	1	1	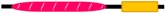
Ricin B/ricin B	0	0	3	0	0	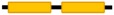
RIP/ricin B/ricin B	0	0	2	0	0	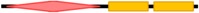

* Subscript specifies the number of domains (t: 1–2; u: 0–2; v: 1 or 4; w: 1–4; x: 0–1; y: 1, 2 or 4; z: 1–3); ^1^ A complete list of all domain combinations is available in Table S2.

## Supplementary Materials

Supplementary materials can be found at www.mdpi.com/1422-0067/18/6/1136/s1.
