# Supplementary material for: Comparative Study of Lectin Domains in Model Species: New Insights into Evolutionary Dynamics
Source: Int J Mol Sci. 2017 May 25;18(6):1136. doi: 10.3390/ijms18061136 (PMC5485960; doi:10.3390/ijms18061136)
Supplement: Supplementary file 1 [file ijms-18-01136-s001.zip › 1-ijms-198054 -supplenentary.pdf]

Comparative Study of Lectin Domains in Model Species: New Insights into Evolutionary Dynamics

Sofie Van Holle [1], Kristof De Schutter [1,2], Lore Eggermont [1], Mariya Tsaneva [1], Liuyi Dang [1] and Els J.M. Van Damme [1,*]

Supplementary Materials:

Table S1. Relationship between the number of lectin genes and genome size, chromosome number and protein-coding transcripts

	Arabidopsis	Soybean	Cucumber	Rice (*japonica*)	Rice (*indica*)
Total number of lectin genes	216	368	146	329	278
Genome size (Mb)	135	1,115	367	385	389
Chromosome number	5	20	7	12	12
Protein-coding transcripts	35,386	88,647	32,528	52,424	56,284
Ratio lectins/genome size	1.60	0.33	0.40	0.85	0.71
Percentage lectins/protein-coding transcripts	0.61	0.42	0.45	0.62	0.49
Number of whole genome duplications	3	3	1	3	3

Table S2. Number of all identified domain combinations containing lectin motifs in the species under study. Numbers between brackets indicate the number of repeats.

Showed in the excel of Supplementary Table S2.

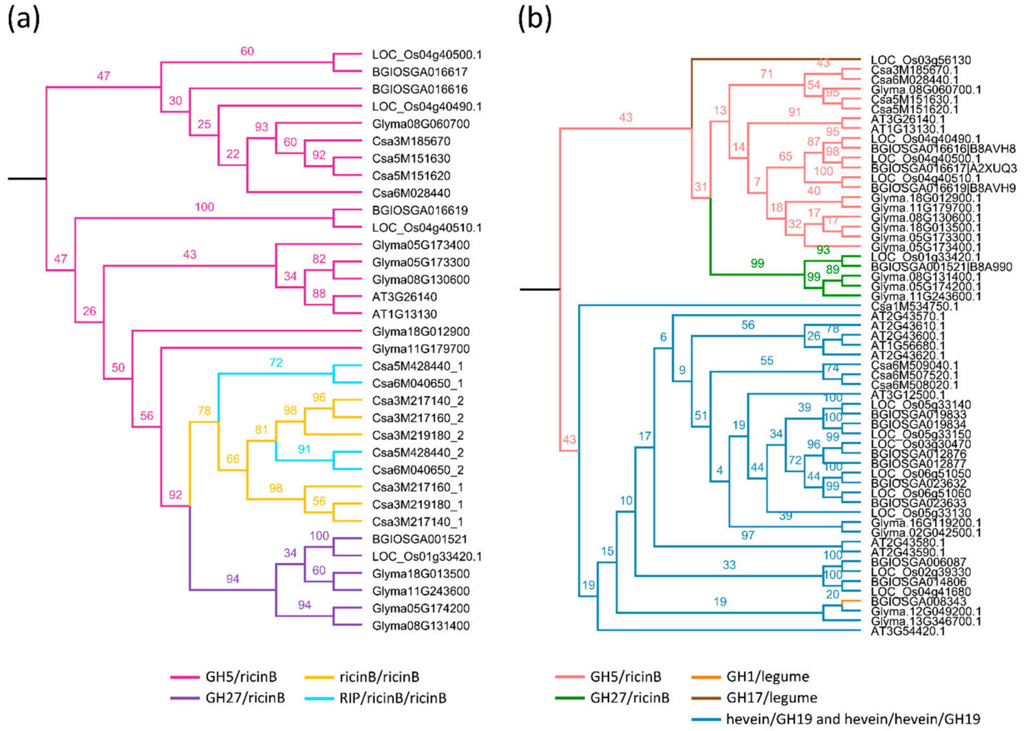

Figure S1. Phylogenetic relationships of all ricin B (**a**) and GH (**b**) domain sequences from *Arabidopsis* (AT), soybean (Glyma), cucumber (Csa), and rice (*japonica*: LOC_Os, *indica*: BGIOSGA). The numbers indicate the bootstrap values and the colored branches correspond to the different domain architectures of the full-length ricin B (**a**) and GH (**b**) protein sequences.

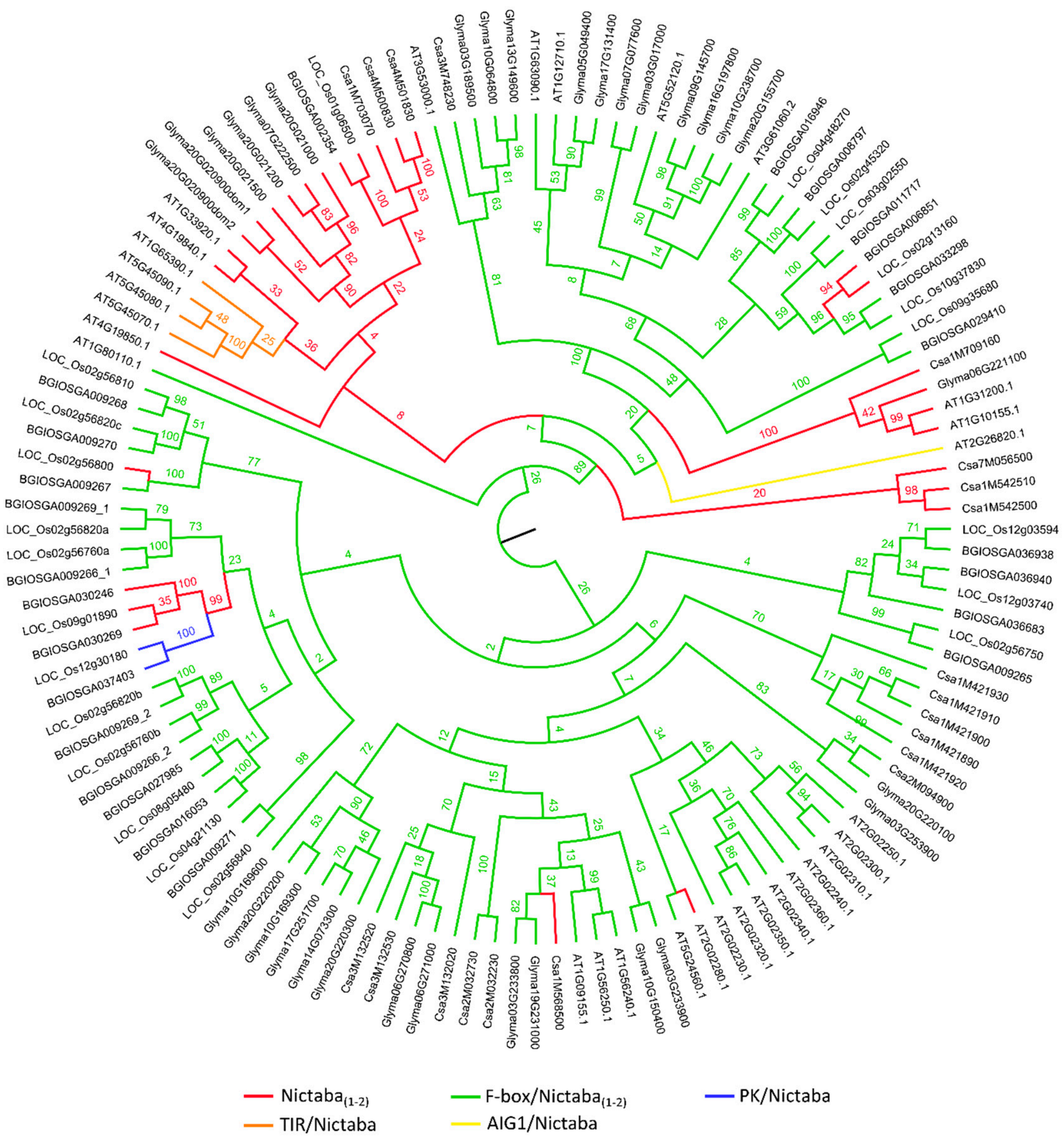

Figure S2. Phylogenetic relationships of the Nictaba domain sequences from *Arabidopsis* (AT), soybean (Glyma), cucumber (Csa), and rice (*japonica*: LOC_Os, *indica*: BGIOSGA). The numbers indicate the bootstrap values and the colored branches correspond to the different domain architectures of the full-length Nictaba protein sequences.

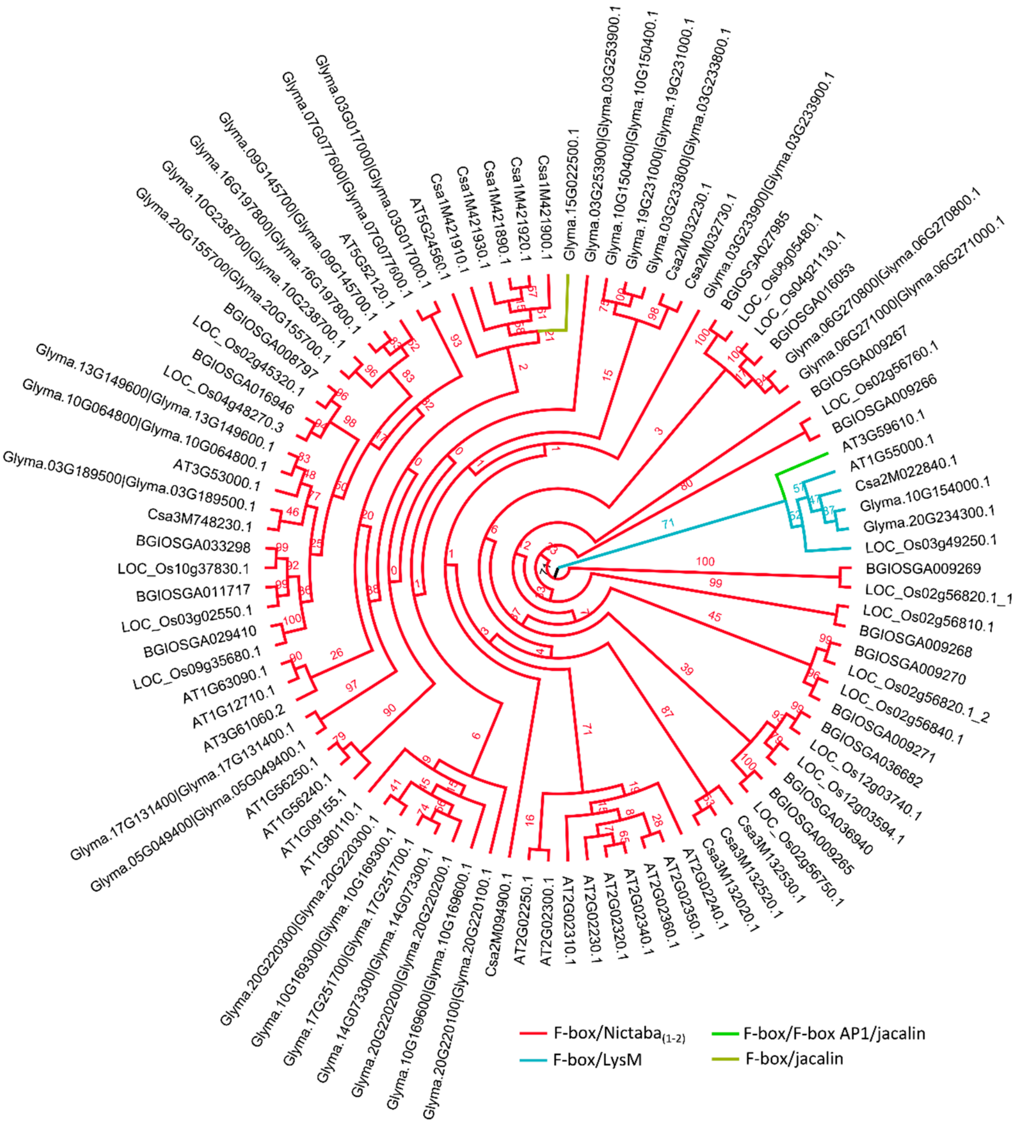

Figure S3. Phylogenetic relationships of the F-box domain sequences from *Arabidopsis* (AT), soybean (Glyma), cucumber (Csa), and rice (*japonica*: LOC_Os, *indica*: BGIOSGA). The numbers indicate the bootstrap values and the colored branches correspond to the different domain architectures of the full-length F-box protein sequences.

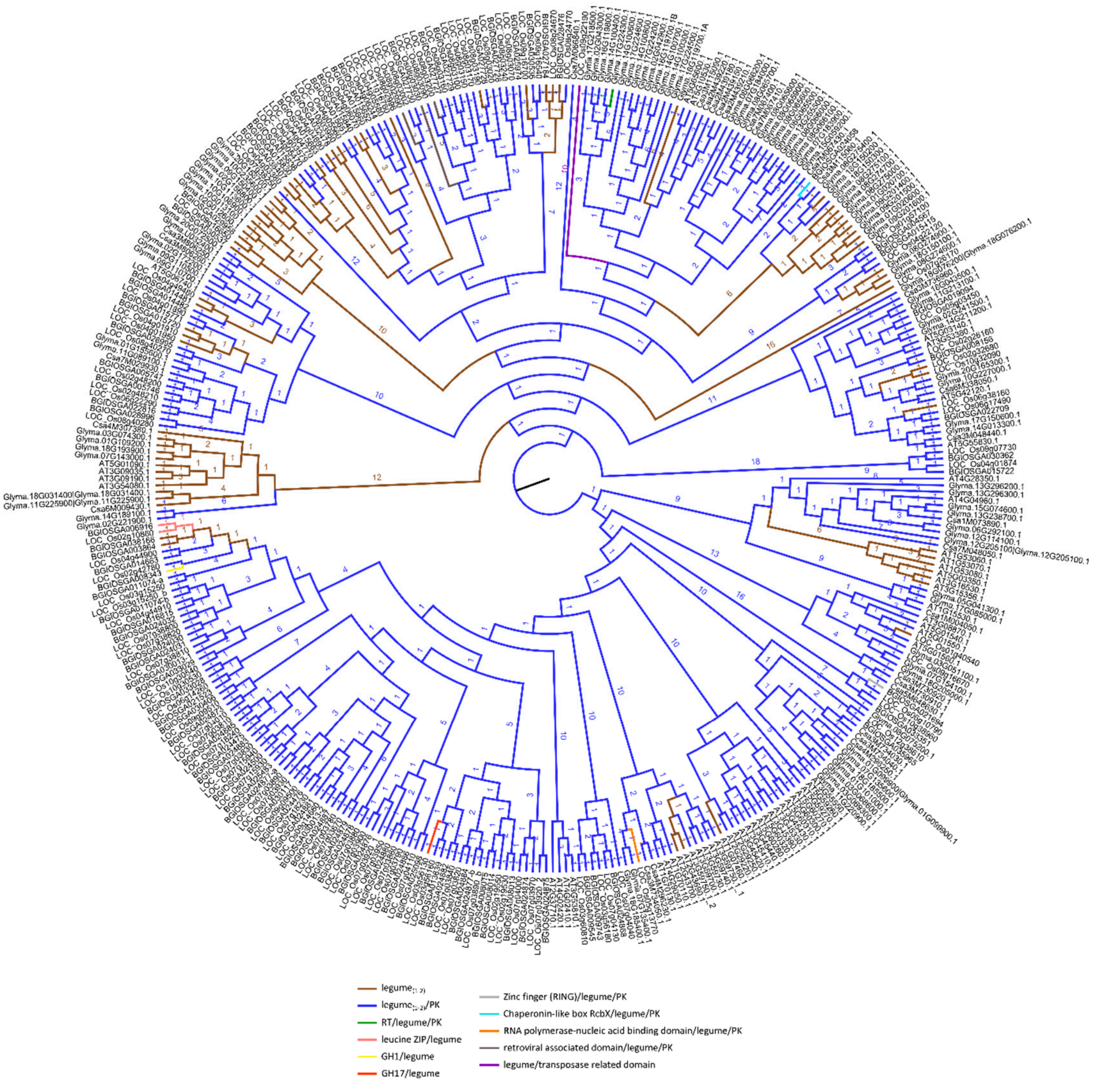

Figure S4. Phylogenetic relationships of the legume lectin domain sequences from *Arabidopsis* (AT), soybean (Glyma), cucumber (Csa), and rice (*japonica*: LOC_Os, *indica*: BGIOSGA). The numbers indicate the bootstrap values and the colored branches correspond to the different domain architectures of the full-length protein sequences.

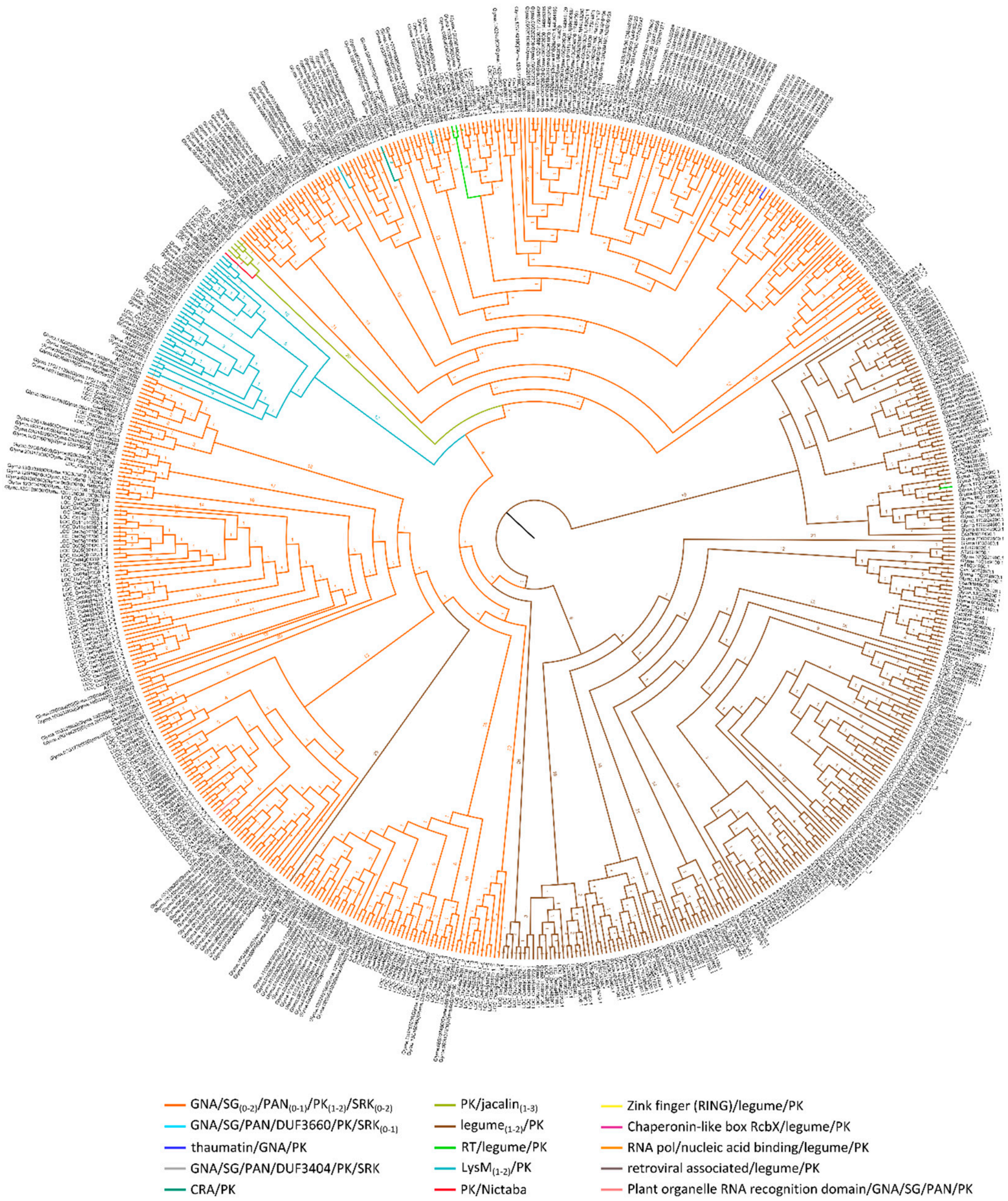

Figure S5. Phylogenetic relationships of the PK domain sequences from *Arabidopsis* (AT), soybean (Glyma), cucumber (Csa), and rice (*japonica*: LOC_Os). The numbers indicate the bootstrap values and the colored branches correspond to the different domain architectures of the full-length PK sequences.

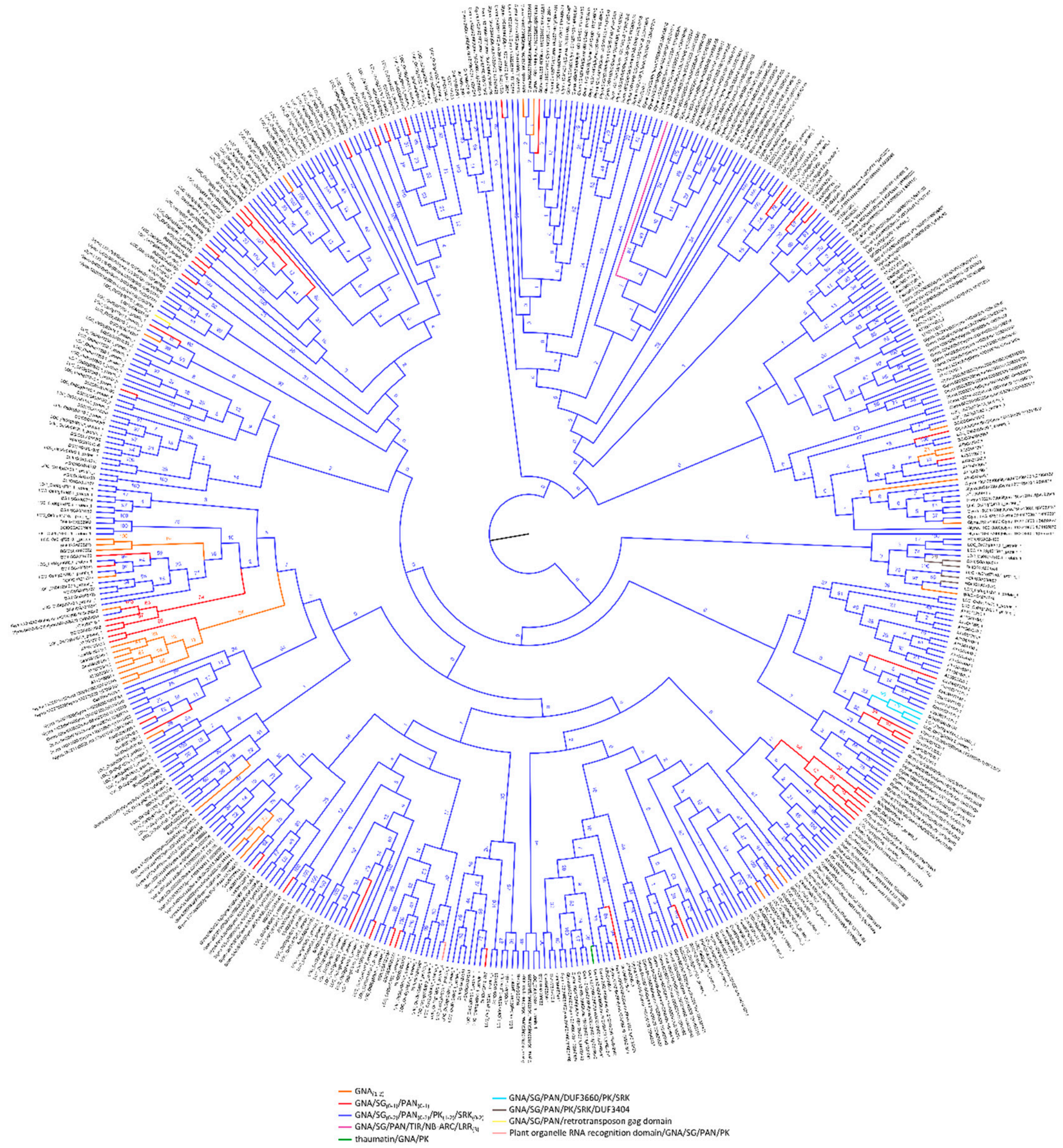

Figure S6. Phylogenetic relationships of the GNA domain sequences from *Arabidopsis* (AT), soybean (Glyma), cucumber (Csa), and rice (*japonica*: LOC_Os, indica: BGIOSGA). The numbers indicate the bootstrap values and the colored branches correspond to the different domain architectures of the full-length GNA lectin sequences.

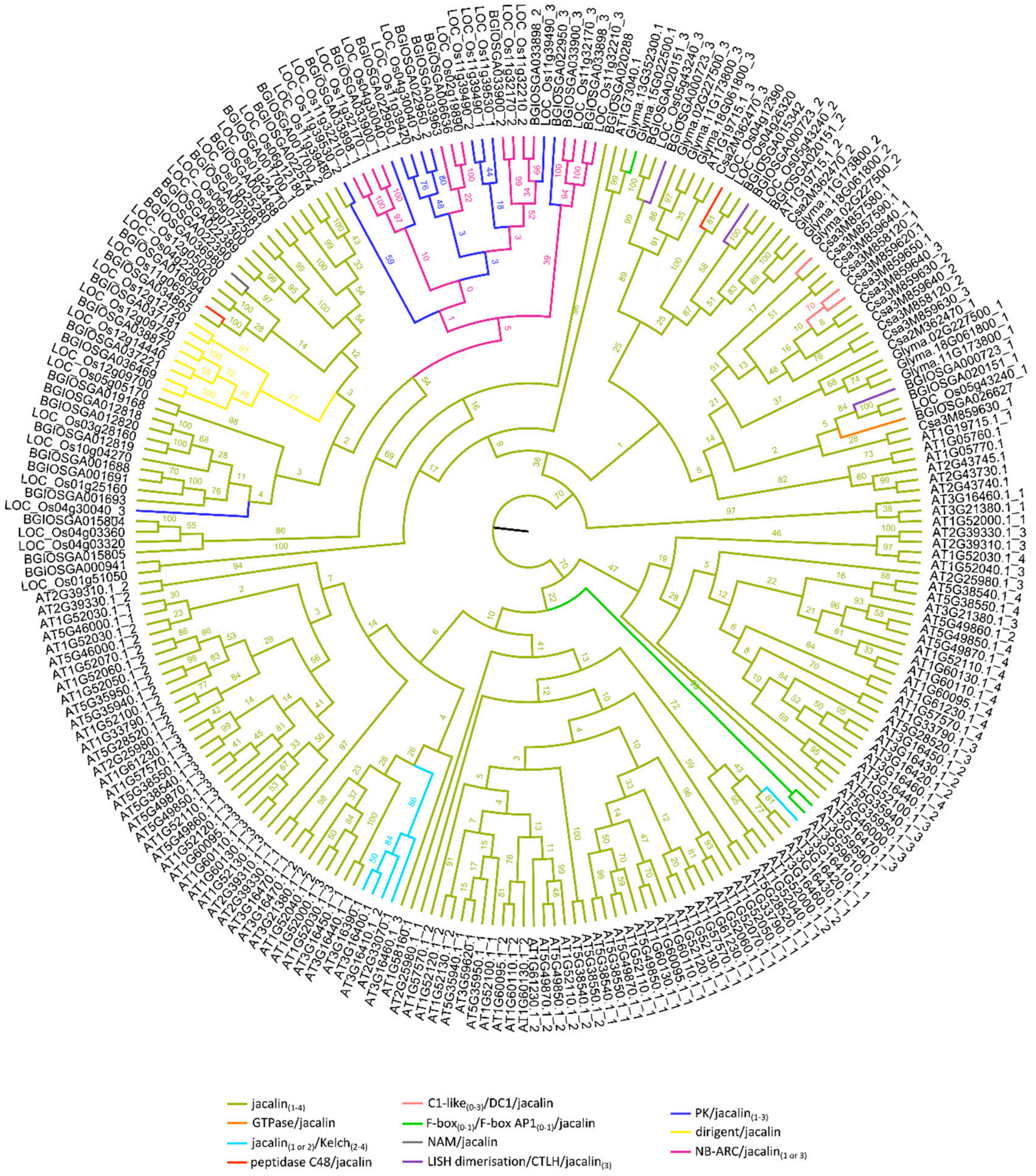

Figure S7. Phylogenetic relationships of the JRL domain sequences from *Arabidopsis* (AT), soybean (Glyma), cucumber (Csa), and rice (*japonica*: LOC_Os, *indica*: BGIOSGA). The numbers indicate the bootstrap values and the colored branches correspond to the different domain architectures of the full-length lectin sequences.

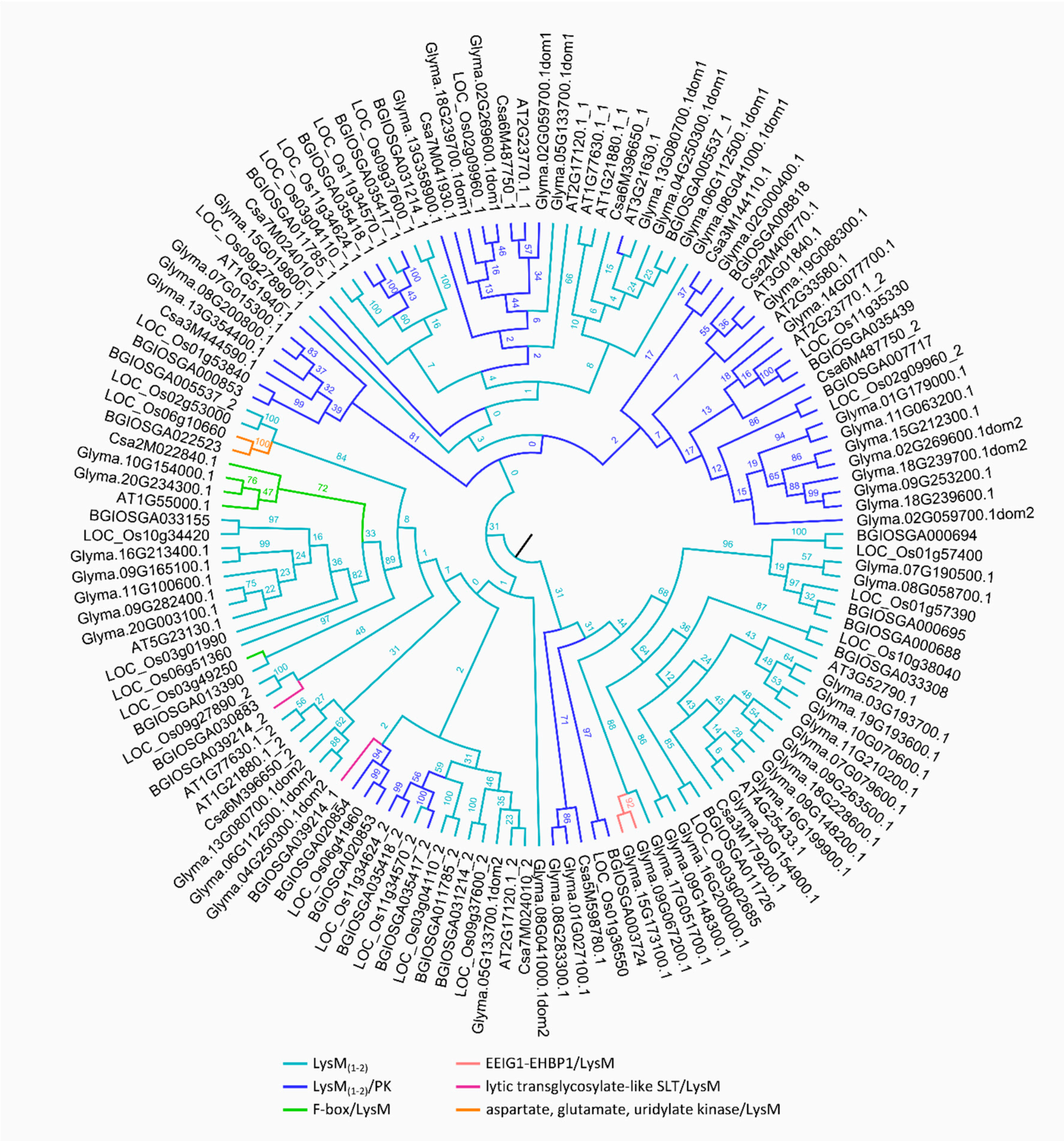

Figure S8. Phylogenetic relationships of the LysM domain sequences from *Arabidopsis* (AT), soybean (Glyma), cucumber (Csa), and rice (*japonica*: LOC_Os, *indica*: BGIOSGA). The numbers indicate the bootstrap values and the colored branches correspond to the different domain architectures of the full-length LysM sequences.

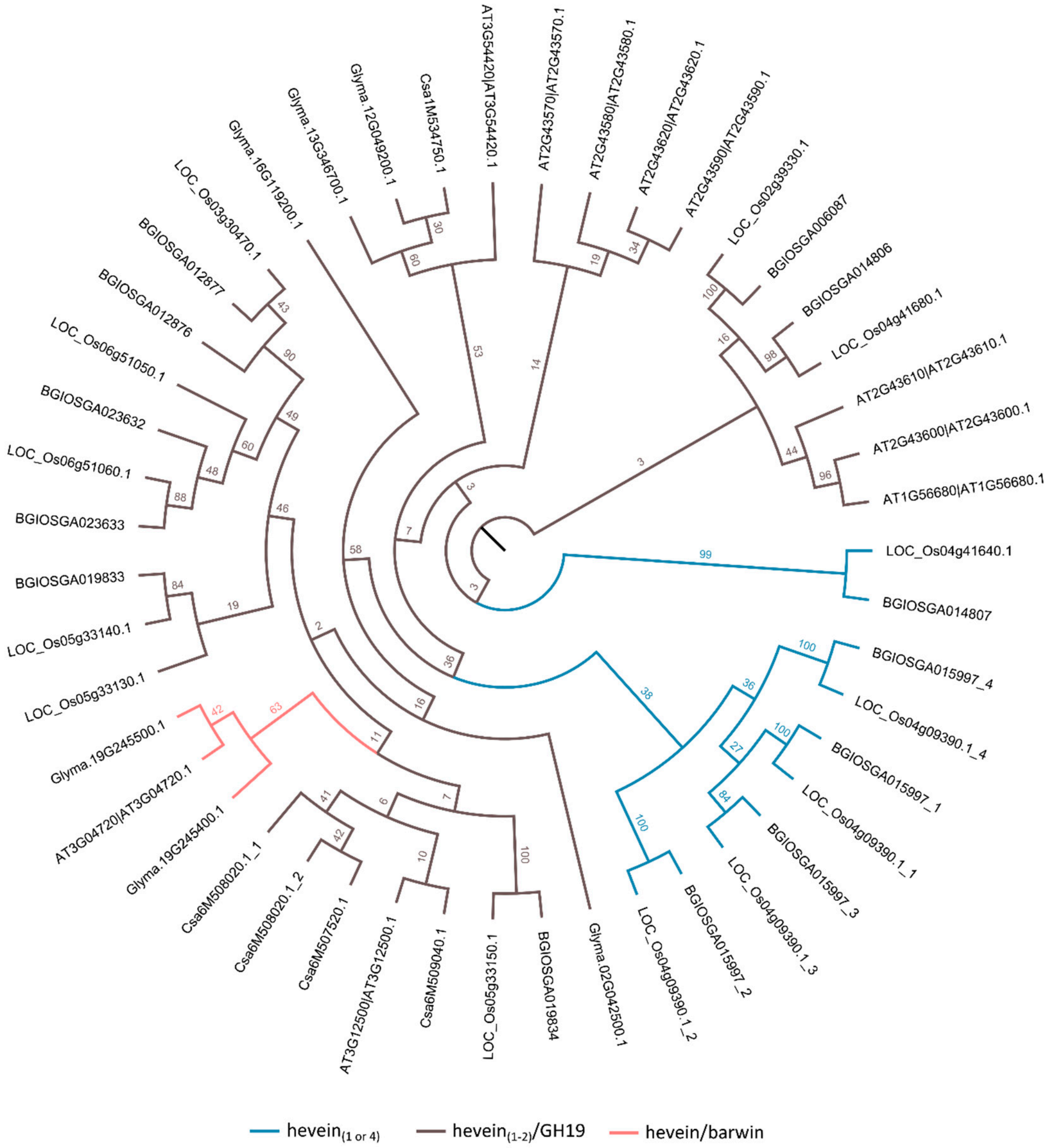

Figure S9. Phylogenetic relationships of the hevein domain sequences from *Arabidopsis* (AT), soybean (Glyma), cucumber (Csa), and rice (*japonica*: LOC_Os, *indica*: BGIOSGA). The numbers indicate the bootstrap values and the colored branches correspond to the different domain architectures of the full-length hevein sequences.
